# Functional Components and Anti-Nutritional Factors in Gluten-Free Grains: A Focus on Quinoa Seeds

**DOI:** 10.3390/foods10020351

**Published:** 2021-02-07

**Authors:** Valentina Melini, Francesca Melini

**Affiliations:** CREA Research Centre for Food and Nutrition, Via Ardeatina 546, I-00178 Rome, Italy; francesca.melini@crea.gov.it

**Keywords:** quinoa, pseudocereals, bioactive compounds, anti-nutrients, phytochemicals, phenolic compounds, saponins, gluten-free, sustainable food

## Abstract

Quinoa (*Chenopodium quinoa* Willd.) has recently received increasing interest from both scientists and consumers due to its suitability in gluten-free diets, its sustainability, and its claimed superfood qualities. The aim of this paper is to systematically review up-to-date studies on quinoa functional components and anti-nutritional factors, in order to define a baseline for food scientists approaching the investigation of quinoa phytochemicals and providing evidence for the identification of healthier sustainable foods. State of the art evaluations of phytochemical contents in quinoa seeds were obtained. It emerged that phenolic compounds are the most investigated functional components, and spectrophotometric methods have been mostly applied, despite the fact that they do not provide information about single components. Saponins are the most studied among anti-nutritional factors. Betalains, tannins, and phytoecdysteroids have been poorly explored. Information on factors affecting the phytochemical content at harvesting, such as quinoa ecotypes, crop geographical location and growing conditions, are not always available. A comprehensive characterization, encompassing several classes of functional components and anti-nutritional factors, is mainly available for quinoa varieties from South America. However, defining a standard of quality for quinoa seeds is still challenging and requires a harmonization of the analytical approaches, among others.

## 1. Introduction

The 2030 Agenda for Sustainable Development of the United Nations highlighted the need for devising sustainable food systems to deliver healthy diets for a growing population [1]. This means giving priority to crops that guarantee an improved efficiency in the use of natural resources and contribute to meeting the requirements of a healthy diet. Within this framework, alternative agricultural crops, such as quinoa, have received great attention.

Quinoa (*Chenopodium quinoa* Willd.) is an annual crop belonging to the Chenopodiaceae family, native to the Andean region in South America. It is resilient to agro-ecological extremes, in terms of soils, rainfall, temperature, and altitude. It can thus grow from sea level up to 4500 m above sea level, and can adapt to frost, drought and salinity [2]. It is mainly grown in Peru, Bolivia, Ecuador, Argentina, Colombia and Chile [3], where it has been used as a staple food for centuries.

Recently, the global demand for quinoa has exploded. As a consequence, the cultivation of quinoa has increased. It has been estimated that in Peru, the export volume of quinoa increased 600 times from 1995 to 2014 [4]. Moreover, quinoa cultivation is also spreading in Northern America, Europe, Asia and Africa [2]. In Europe, there were no cultivated areas until 2008, while in 2015 quinoa cultivation regions reached 5000 ha.

Seeds are the main part of the quinoa plant used in human nutrition. They can be consumed whole, similar to rice; puffed, to make breakfast cereals; ground to flour, to prepare baked products; or fermented, to make beer or “chicha”, a traditional alcoholic beverage of Southern and Central America. The leaves have also been used in human nutrition, similar to other leafy vegetables such as spinach [5].

Quinoa has a high nutritional value and is adequate in normal and special diets. It is suitable for gluten-free (GF) diets, not only because it lacks gluten, but also because, as a whole grain, it contributes to overcoming some nutritional inadequacies of GF products [6]. It is, in fact, a source of dietary fiber [7], vitamins and minerals [8]. Quinoa seeds also provide all essential amino-acids, in contrast to cereals [9]. This pseudocereal is thus also recommended for vegetarian and vegan diets.

In Western countries, quinoa is presumed to be a “superfood”. There is no official definition of “superfoods”, nor guidelines about traits and properties a food should have to be considered a “superfood” [10]; however, commonly, a superfood is claimed to represent foods that contain high amounts of antioxidants, vitamins and/or minerals, and they are thus perceived as healthier than equivalent alternatives.

The aim of this paper is to systematically review up-to-date studies on the content of functional components and anti-nutritional factors in quinoa seeds, so as to obtain a baseline for food scientists approaching the investigation of quinoa phytochemicals and providing evidence for the identification of healthier sustainable foods. A systematic literature search of studies investigating the functional components and anti-nutritional factors in quinoa seeds was carried out. Information on hydrophilic and lipophilic phytochemicals, anti-nutrients, and analytical procedures applied for the determination thereof, were gathered and discussed. Based on the above, knowledge gaps and the need for a deeper understanding of the quinoa phytochemical profile are finally presented.

## 2. Materials and Methods

### 2.1. Literature Search Strategy

A systematic literature search for studies on planned topics and published in peer-reviewed journals was carried out from September to October 2020 on SCOPUS database. A five-year time limit was set for electronic search, in order to retrieve only very up-to-date literature. The detailed search strategy is reported in Table 1. References were collated in the reference management software Mendeley (Mendeley Desktop Version 1.19.4, Mendeley Ltd., London, UK). Microsoft^®^ Office^®^ Excel 2010 software was used to collect and process the data.

### 2.2. Study Selection Process and Including and Excluding Criteria

The authors independently screened publications using the following criteria for inclusion. Studies published in languages other than English were excluded. Only research articles reporting the composition of quinoa seeds in terms of functional and anti-nutritional components were included.

Duplicate papers were excluded. Screening of titles, abstracts and full-texts resulted in further exclusion of papers falling beyond the scope of this review. The reference list of publications selected for review was also checked in order to identify studies relevant for review.

A PRISMA (Preferred Reporting Items for Systematic reviews and Meta-Analyses) flow diagram of the study search strategy and selection process was developed.

### 2.3. Data Extraction Criteria

Data extracted for inclusion in the review referred to: (i) functional and/or anti-nutritional component group; (ii) functional and/or anti-nutritional component sub-group; (iii) analytical method(s) used for the determination of targeted analyte(s); (iv) quinoa sample origin; (v) quinoa seed color and ecotype(s).

## 3. Results and Discussion

### 3.1. PRISMA Flow Diagram of the Search Strategy and Selection Process

**Figure 1:**
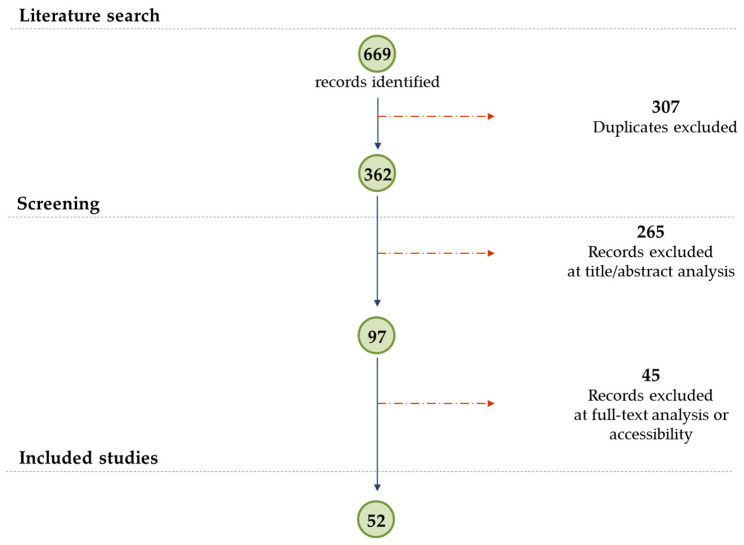
PRISMA (Preferred Reporting Items for Systematic reviews and Meta-Analyses) flow diagram of the study search strategy and selection process.

As shown in the PRISMA flow diagram (Figure 1), 669 publications were retrieved from a search in the SCOPUS database. A total of 307 duplicates were removed and 362 papers were screened for title and abstract. The process resulted in the consolidation of 97 papers that were eligible for text screening. A total of 45 papers were excluded on the basis of the full-text accessibility and analysis. A total of 52 papers were finally selected for analysis in the present review.

### 3.2. Distribution of Included Studies by Functional/Anti-Nutritional Component Class and Sample Geographical Origin

**Figure 2:**
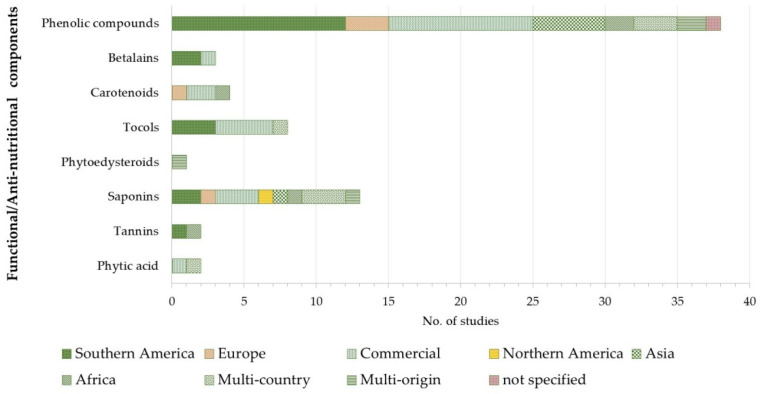
Distribution of included studies by functional/anti-nutritional component class and sample geographical origin.

The analysis of the studies included in this systematic review showed that phenolic compounds are the most investigated functional components in quinoa seeds (Figure 2). A total of 38 studies determined phenolic compound content and/or profile. Among them, twelve studies investigated quinoa samples from South America, five studies were carried out on quinoa grown in Asia, and ten studies analyzed samples retrieved from the market, whose origin was not always reported by the Authors.

Among phenolic compound sub-groups, flavonoids have been largely studied. They have been mainly investigated in quinoa from South America and Asia. On the other hand, flavonols and isoflavones have been poorly studied. The hydrophilic betalains were reported by only three studies. Among lipophilic functional components, tocols are the most investigated, followed by carotenoids. Their content was reported in eight and four studies, respectively.

As far as anti-nutritional factors are concerned, seventeen studies were included in the systematic review. Saponins are the most studied anti-nutrients (Figure 2). This might be due to the fact that they contribute to the bitterness of quinoa and thus play a key role in quinoa appreciation by consumers. In contrast, tannins and phytic acid have been poorly studied.

As regards the geographical origin of studied samples, quinoa grown in South America is the most investigated, and a more comprehensive characterization, encompassing several classes of functional components and anti-nutritional factors, is available.

### 3.3. Functional Components in Quinoa Seeds

Functional components include a variety of molecules that can modulate one or more metabolic processes in the human body, so as to present a wide range of probable effects.

They can be hydrophilic (i.e., phenolic compounds and betalains) or lipophilic compounds (i.e., carotenoids, tocols, and phytoecdysteroids). Their structural diversity and content, as emerged from the selected studies, are presented and discussed in the following sections.

#### 3.3.1. Phenolic Compounds

Phenolic compounds (PCs) are plant secondary metabolites that include a diversity of chemical structures sharing the presence of one or more hydroxyl groups on aromatic ring(s). They can be found in free form, esterified, or bound to cell wall components [11].

They are ubiquitous in plants, and hence, also in plant-derived foods. The dietary intake of PCs promotes health and well-being. These components can, in fact, modulate the metabolism of carbohydrates and lipids, improve pancreas β-cell function and stimulate insulin secretion. Hence, they play an important role in attenuating hyperglycemia, dyslipidemia and insulin resistance [12]. They also have antioxidant, anti-inflammatory and anti-proliferative activity, thus they contribute to preventing the onset of non-communicable diseases [13].

Among dietary phenolics, flavonoids and phenolic acids are the most abundant. They have been greatly identified in quinoa seeds. Characterization of phenolic compounds in quinoa seeds was performed by spectrophotometric or chromatographic methods. The former enables one to quantify the total phenolics or specific sub-groups, such as total flavonoids, while the latter allows obtaining the profile of single components.

##### Quantitation of Phenolic Compounds by Spectrophotometric Assays

Phenolic compounds in quinoa seeds have been mainly quantitated by spectrophotometric methods (Table 2). Quantitation is expressed as total phenolic content (TPC), free phenolic content (FPC) and bound phenolic content (BPC).

The Folin–Ciocalteu assay is mostly used. It is simple and relatively rapid. Moreover, it requires reagents rather inexpensive. The Prussian Blue assay is also used to determine the content of phenolic compounds in quinoa seeds.

As regards quinoa seeds grown in Africa, samples from Morocco and Egypt were analyzed. The highest TPC value—202 mg gallic acid equivalents (GAE) 100 g^−1^ dry matter (dm)—was found in a sample belonging to the Q52 genotype, grown from mid-November 2016 to April 2017 in the area of South Sinai (Egypt) [14]. Lower values were found for the genotypes kvlsra2, kvl-sra3, Q37 and Regalona [14]. The latter showed the lowest content (Table 2). Since the five genotypes were grown in the same geographical area and during the same season, differences in phenolic content were likely due to differences in genotype. TPC values lower than those reported for quinoa from Egypt were found in quinoa raw seeds of the Titicaca and Puno varieties grown in Morocco (Table 2) [15]. The effect of polishing on TPC was also investigated, and it was found that processing decreased the TPC by approximately 60% in Titicaca seeds, while no statistical differences were observed in the Puno variety (Table 2). 

Phenolic compounds were also detected in quinoa samples originating from different areas of Asia, specifically China, India and Korea. As regards quinoa cultivated in China, white and pigmented varieties were analyzed. Han et al. studied the Chinese quinoa cultivar, Jinli-1, with non-pigmented seeds. Phenolic compounds (TPC, FPC and BPC) and flavonoids (total flavonoid content (TFC), flavonoids in extractable forms (FFC) and bound flavonoid content (BFC)) were determined within the wider scope of understanding the extent to which the milling process can affect the content of these components [16]. Indeed, it is known, that phenolic compounds are located in the grain outer layers, and the degree of milling (DOM) impacts their content. As regards phenolic compound quantification in raw seeds, the TPC was 200.40 mg GAE 100 g^−1^ dm, with free phenolics representing the main fraction (Table 2). Upon milling (DOM 27.23%), both free and bound phenolics decreased significantly (Table 2). However, after processing, the free fraction was still the most abundant. In contrast, Li et al. observed that the BPC was higher than FPC in an improved quinoa variety from Peruvian altiplano, grown in China, referred to as Jiaqi-1 [17]. In detail, BPC was five-fold higher than FPC (Table 2). In quinoa samples from the Shanxi region (China), Liu et al. found that the FPC was higher than the BPC in white quinoa seeds (380 vs. 50 mg GAE 100 g^−1^ dm), which is in keeping with Han et al. [16]. However, in pigmented quinoa, phenolics were mainly in the bound form (Table 2) [18]. Moreover, TPC in pigmented varieties was two to three-fold higher than in white varieties. Differences in phenolic content among quinoa samples grown in China might be due to differences in genotypes, as well as to different pedoclimatic conditions. 

The phenolic content levels observed in China were significantly higher than those observed in quinoa samples from India and Korea. The white ecotype of Royal variety quinoa, grown in India, was analyzed by Kaur et al. [19] who found a TPC of 43.2 mg GAE 100 g^−1^ dm in raw seeds (Table 2). The effect of soaking, dehulling and germinating was also investigated, and a lower TPC was found in soaked and dehulled seeds, while an almost three-fold increase in TPC occurred in germinated seeds. The lowest TPC value (14.37 mg GAE 100 g^−1^ dm) among quinoa seeds from Asia was found in quinoa samples grown in Korea (Table 2) [20].

As regards quinoa grown in Europe, samples from Finland and Serbia were analyzed. In raw quinoa grown in Finland, great variability in the TPC was observed. Mattila et al. reported that the TPC was 181 and 597 mg GAE 100 g^−1^ dm (Table 2), when the Folin–Ciocalteu and the Prussian Blue assays were used, respectively [21]. These values significantly decreased after pearling, since phenolics are concentrated in the outer layers of the quinoa seeds. Multari et al. [22] found the values of phenolic compounds in quinoa samples from Finland to be lower than those determined by Mattila et al., with the free faction accounting for only 10% of the total phenolics (Table 2). In two cultivars (i.e., Puno and Titicaca) adapted to the European climatic conditions and grown in Serbia at the end of the 2016/2017 growing season, the free phenolic fraction was more than two-fold higher than the bound fraction [23], and was far higher than that in quinoa grown in Finland [22]. 

As regards quinoa from South America, samples from Peru, Chile, Brazil, Argentina and Colombia were studied. Defining a range of values for quinoa grown in a geographical area is challenging because of differences in the studied parameters. FPC values were determined in quinoa grown in Peru and Chile [24,25,26]. Some of the quinoa cultivars grown in Peru showed values higher than those in Chile (Table 2). Bound phenolics values were determined in samples from Peru and Chile as well [24,25]. In the sample from Peru, these were comparable to the free fraction [24], while in quinoa from Chile they were three to five-fold lower (Table 2) [26]. 

Most studies reported only TPC, and some mainly targeted investigating the effect of processing [27,28,29,30,31,32]. As regards raw seeds, TPC values ranged from approximately 39 to 490 mg GAE dm (Table 2). The highest TPC was observed in quinoa seeds from Peru by Paucar-Menacho et al. (489.78 mg GAE 100 g^−1^ dm) [27]. The lowest was found in quinoa samples grown in Buenos Aires province (Argentina) [29,30,31].

As regards the effect of quinoa processing on TPC, Nickel et al. found that toasting significantly decreased the TPC of quinoa from Brazil (variety BRS-Piabiru), while washing and cooking increased it. Cooking of washed grains, at atmospheric pressure and under pressure, increased TPC as well (Table 2). This trend was possibly due to a softening or disintegrating effect on the grain tissues, promoting the release of phenolics from the food matrix, as observed in red rice samples [33]. Quinoa roasting, germination and fermentation also increased the TPC values (Table 2) [29,30,31]. Buitrago et al. studied the effect of drying treatments (i.e., freeze- and air-drying) on the TPC in quinoa grown in Colombia [32]. It was observed that the TPC was higher in freeze-dried samples than in the air-dried ones (Table 2). The lower amount in the air-dried samples was likely due to phenolic compounds binding to proteins and hindering extraction and/or determination by colorimetric methods.

Some authors studied quinoa samples of different geographical origins [34,35,36]. Samples from Peru, the United States and Korea were studied by Lee et al. [34] and Park et al. [35]. But on average the former found TPC values that were 30-fold higher than the latter (Table 2). It can be assumed that such variability was due to sample intrinsic factors and to the applied extraction procedure. Sobota et al. studied 25 cultivars of yellow-coated quinoa originating from Argentina, Chile, Denmark, Poland, and the USA [36]. The highest and lowest TPC values were found in two Chilean varieties with values of 1060 and 710 mg 100 g^−1^ dm, respectively (Table 2).

Phenolic quantitation has been often performed on quinoa seeds retrieved from the market. These studies provide valuable data to assess the nutritional quality of quinoa that is eaten by consumers, to estimate the dietary intake of bioactive compounds or to evaluate the nutritional status of the population. Unfortunately, the lack of information on the quinoa genotype, growing location and condition or post-harvest processing do not contribute to gaining information that might reorient quinoa production towards that of a higher quality. Commercial white, red and black quinoa samples were analyzed [37,38,39]. The lowest values for FPC in white quinoa seeds were found by Pellegrini et al. [38], while the highest values were observed by Škrovánková et al. [37]. As regards pigmented quinoa varieties, FPC values were lower than the white samples in the study by Škrovánková et al. [37], which is comparable to those determined by Pellegrini et al. [38], and higher that those determined by Tang et al. [39] (Table 2). Navarro del Hierro et al. determined FPC in extracts of a commercial quinoa sample, obtained by using three different extracting solvents [40]. It was found that a mixture of ethanol:water gave the highest yield (Table 2). Diaz-Valencia et al. studied both commercial quinoa samples and quinoa seeds grown in South America [41]. They found comparable FPC values in white and red quinoa, in keeping with Pellegrini et al. [38], while values ranging from 55.5 to 95.9 mg GAE 100 g^−1^ dm were reported for black quinoa (Table 2). In a commercial sample available on the USA market, a low TPC value (1.37 mg GAE 100 g^−1^ dm) was found by Balakrishnan et al. [42]. As regards quinoa samples available on the Italian market, Laus et al. observed a TPC of 1.8 µmol GAE g^−1^ dm [43], while Rocchetti et al. found that the TPC was 67.7 mg GAE 100 g^−1^ dm (Table 2) [44]. They also determined the phenolic profile by UHPLC-MS and observed that phenolic acids were the most abundant, followed by flavanols. Hur et al. analyzed quinoa samples purchased in Seoul (Korea) [45]. They determined the TPC by spectrophotometric methods, and found that it was 4.1 mg GAE 100 g^−1^ in the ethanolic extract (Table 2).

Specific sub-groups of phenolic compounds, such as flavonoids and flavonols, have also been quantified by spectrophotometric methods and their content was expressed as total flavonoid content (TFC) or total flavonol content. Their free and bound fractions were also quantified.

Flavonoids have a distinctive benzo-γ-pyrone skeleton and occur as aglycones (free form), glycosides and methylated derivatives (bound form). They comprise flavonols, flavan-3-ols, flavones, isoflavones, flavanones, anthocyanidins, and chalcones. Almost all groups of flavonoids act as antioxidants and are thus responsible for a number of health benefits [46]. Their determination is, thus, important for understanding the extent to which the intake of quinoa can contribute to the intake of functional components.

Several studies have reported on the TFC in quinoa seeds. Saad-Allah et al. found TFC values ranging from 127.1 to 288.8 µg g^−1^ dm in quinoa from Egypt (Table 2) [14]. As for quinoa grown in Asian countries, it was not possible to define a range for flavonoid content, as different flavonoids were used as standards for the calibration. Lim et al. reported 45.88 mg Rutin Equivalent (RE) 100 g^−1^ dm for TFC in seeds from Korean crops (Table 2) [20]. Both free and bound flavonoids were reported for samples from China (Table 2) [16,18], but these values are not comparable since rutin and quercetin were used as standards for the calibration. In the Chinese quinoa cultivar Jinli-1, the FFC and BFC values were 147.95 and 76.61 mg Catechin Equivalents (CE) 100 g^−1^ dm, respectively (Table 2) [16]. As regards flavonoids in white and pigmented genotypes of quinoa from the Shanxi province (China), the highest values of FFC, BFC and TFC were observed in red quinoa seeds (Table 2) [18]. TFC was also detected in white quinoa seeds (Royal variety) grown in India and a content of 11.4 mg quercetin equivalent (QE) 100 g^−1^ was found (Table 2) [19]. 

Flavonoids in quinoa from Europe were only reported by Stikić et al. who studied samples of Puno and Titicaca cultivars grown in Serbia [23]. A higher content of total flavonoids (FFC+BFC) was observed in Titicaca seeds than in Puno seeds (Table 2). FFC were not significantly different between both cultivars, while the bound form was higher in the Puno cultivar than in Titicaca seeds (Table 2).

Flavonoids in quinoa samples from Peru, the USA and Korea were studied by Lee et al. [34] and Park et al. [35]. As observed for TPC, TFC values in the samples studied by Lee et al. were higher than in the quinoa seeds analyzed by Park et al. (Table 2). The former found values ranging from 160 to 218 mg QE 100 g^−1^ dm, with the sample from Peru showing the highest TFC (Table 2) [34]. Park et al. observed TFC values ranging from 11.51 to 20.91 mg QE 100 g^−1^ dm, and identified the highest TFC in the sample from Korea and the lowest in the quinoa sample imported from Peru (Table 2) [35]. As regards samples from South America, some of the quinoa cultivars grown in Chile showed values higher than those in Peru (Table 2) [25,26]. The effect of processing on flavonoid content was investigated in quinoa samples grown in Buenos Aires province (Argentina) [29,31]. TFC values increased after processing (Table 2). The effect of different drying treatments (i.e., freeze- and air-drying) on TFC was also studied in quinoa grown in Colombia [32]. TFC was higher in freeze-dried samples than in air-dried seeds, with values of 504 and 154 mg QE 100 g^−1^ dm, respectively [32]. 

Flavonoids in commercial samples have been under-investigated. TFC in the ethanolic extract of quinoa samples purchased in Seoul (Korea) was 1.3 mg QE 100 g^−1^ [45]. Tang et al. determined the FFC and BFC values of white, red and black quinoa samples purchased in Ontario and found that red quinoa showed the highest TFC (≈175 mg CE 100 g^−1^ dm) (Table 2) [39]. Two different hydrolytic conditions (alkaline and acid hydrolysis) were also applied to determine BFC. It was found that hydrolysis in acid conditions, enabled the attainment of the highest flavonoid content.

Flavonols were also detected in quinoa seeds, but only one study has been published in the last five-years [24]. These components have a 3-hydroxy-2-phenylchromen-4-one skeleton and can occur in free and bound form. In quinoa samples from Peru, free flavonol content ranged between 30 and 113 mg QE 100 g^−1^ fw, while bound flavonols varied from 16 to 147 mg QE 100 g^−1^ fw (Table 2).

**Table 2:** Phenolic compounds in quinoa seeds quantified by spectrophotometric methods.

Analyte	Analyte Content	Quinoa Sample Origin	Reference
TPC	TPC (Titicaca, raw): 105.85 ^1^ TPC (Puno, raw): 31.67 ^1^ TPC (Titicaca, polished): 67.86 ^1^ TPC (Puno, polished): 26.31 ^1^	Africa, Morocco	Mhada et al. [15]
TPC	TPC: 66–202 ^1^	Africa, Egypt	Saad-Allah et al. [14]
FPC, BPC	FPC (raw): 162.90 ^1^ BPC (raw): 73.50 ^1^ TPC (raw): 200.40 ^1^ FPC (milled): 111.51 ^1^ BPC (milled): 25.85 ^1^ TPC (milled): 137.36 ^1^	Asia, China	Han et al. [16]
FPC, BPC	FPC: 205 ^1^ BPC: 1180 ^1^	Asia, China	Li et al. [17]
FPC, BPC, TPC	FPC (white quinoa): ≈380 ^1^ FPC (red quinoa): 609.74 ^1^ FPC (black quinoa): 506.70 ^1^ BPC (white quinoa): ≈70 ^1^ BPC (red quinoa): 799.80 ^1^ BPC (black quinoa): 563.07 ^1^ TPC (white quinoa): ≈515 ^1^ TPC (red quinoa): 1409.54 ^1^ TPC (black quinoa): 1069.77 ^1^	Asia, China	Liu et al. [18]
TPC	TPC (raw): 43.2 ^1^ TPC (soaked): 31.1 ^1^ TPC (dehulled): 34.6 ^1^ TPC (germinated): 101.2 ^1^	Asia, India	Kaur et al. [19]
TPC	TPC: 14.37 ^1^	Asia, Korea	Lim et al. [20]
TPC	TPC (unpearled, FC): 181 ^1^ TPC (pearled, FC): 20 ^1^ TPC (unpearled, PB): 597 ^1^ TPC (pearled, PB): 62 ^1^	Europe, Finland	Mattila et al. [21]
FPC, BPC	FPC: 3.47 ^1^ BPC: 29.7 ^1^	Europe, Finland	Multari et al. [22]
FPC, BPC	FPC: 56.627–67.856 ^1^ BPC: 25.354–26.667 ^1^	Europe, Serbia	Stikić et al. [23]
FPC, BPC	FPC: 123–341 ^2^ BPC: 128–452 ^2^	South America, Peru	Abderrahim et al. [24]
FPC	FPC: 358 ^1^	South America, Peru	Drzewiecki et al. [26]
FPC, BPC	FPC: 97–164 ^1^ BPC: 16–53 ^1^	South America, Chile	Vega-Gálvez et al. [25]
TPC	TPC: 489.78 ^1^	South America, Peru	Paucar-Menacho et al. [27]
TPC	TPC (raw): 97.60 ^1^ TPC (washed): 116.77 ^1^ TPC (washed, hydrated): 96.32 ^1^ TPC (washed, cooking): 110.65 ^1^ TPC (washed, cooking pressure): 127.54 ^1^ TPC (washed, toasting): 58.63 ^1^	South America, Brazil	Nickel et al. [28]
TPC	TPC (raw): 40.15 ^1^ TPC (roasted): ↑ 18–60%	South America, Argentina	Carciochi et al. [29]
TPC	TPC (raw): 39.3 ^1^ TPC (fermented): ↑ 46%	South America, Argentina	Carciochi et al. [30]
TPC	TPC (raw): 39.29 ^1^ TPC (malted): 79.04 ^1^	South America, Argentina	Carciochi et al. [31]
TPC	TPC (freeze-dried samples): 1500 ^1^ TPC (air-dried samples): 700 ^1^	South America, Colombia	Buitrago et al. [32]
TPC	TPC (Peru): 500 ^1^ TPC (USA): 470 ^1^ TPC (Korea): 384 ^1^	Multi-country (Peru, USA and Korea)	Lee et al. [34]
TPC	TPC (Peru): 15.33 ^1^ TPC (USA): 16.28 ^1^ TPC (Korea): 14.50 ^1^	Multi-country (Peru, USA and Korea)	Park et al. [35]
TPC	TPC: 710–1060 ^1^	Multi-country (Argentina, Chile, Denmark, Poland, and USA)	Sobota et al. [36]
FPC	FPC (ethanol:water): 2.62 ^1^ FPC (ethanol): 0.76 ^1^ FPC (water): 0.75 ^1^	Commercial	Navarro del Hierro et al. [40]
FPC	FPC (white): 226.1 ^1^ FPC (red): 97.3 ^1^ FPC (black): 100.5 ^1^	Commercial	Škrovánková et al. [37]
FPC	FPC (white): 75.297–87.584 ^2^ FPC (red): 85.353 ^2^ FPC (black): 79.097 ^2^	Commercial	Pellegrini et al. [38]
FPC	FPC (white): ≈200 ^1^ FPC (red): ≈490 ^1^ FPC (black): ≈510 ^1^	Commercial	Tang et al. [39]
FPC	FPC (white): 59.6–66.4 ^1^ FPC (red): 61.1–65.4 ^1^ FPC (black): 55.5–95.9 ^1^	Multi-origin (Commercial + South America, Peru)	Diaz-Valencia et al. [41]
TPC	TPC: 1.37 ^1^	Commercial	Balakrishnan et al. [42]
TPC	TPC: ≈1.8 ^3^	Commercial	Laus et al. [43]
TPC	TPC: 67.7 ^1^	Commercial	Rocchetti et al. [44]
TPC	TPC: 4.1 ^1^	Commercial	Hur et al. [45]
TFC	TFC: 127.1–288.8 ^4^	Africa, Egypt	Saad-Allah et al. [14]
TFC	TFC: 45.88 ^5^	Asia, Korea	Lim et al. [20]
FFC, BFC, TFC	FFC: 147.95 ^6^ BFC: 76.61 ^6^ TFC: 224.56 ^6^	Asia, China	Han et al. [16]
FFC, BFC, TFC	FFC (white): ≈140 ^5^ FFC (red): ≈240 ^5^ FFC (black): ≈200 ^5^ BFC (white): ≈35 ^5^ BFC (red): ≈160 ^5^ BFC (black): ≈140 ^5^ TFC (white): ≈175 ^5^ TFC (red): ≈400 ^5^ TFC (black): ≈350 ^5^	Asia, China	Liu et al. [18]
TFC	TFC (raw): 11.4 ^7^	Asia, India	Kaur et al. [19]
FFC, BFC, TFC	FFC (Puno): 78.49 ^7^ FFC (Titicaca): 87.32 ^7^ BFC (Puno): 11.21 ^7^ BFC (Titicaca): 7.04 ^7^ TFC (Puno): 89.71 ^7^ TFC (Titicaca): 93.45 ^7^	Europe, Serbia	Stikić et al. [23]
TFC	TFC (Peru): 218 ^7^ TFC (USA): 176 ^7^ TFC (Korea): 160 ^7^	Multi-country (Peru, USA and Korea)	Lee et al. [34]
TFC	TFC (Peru): 11.51 ^7^ TFC (USA): 13.24 ^7^ TFC (Korea): 20.91 ^7^	Multi-country (Peru, USA and Korea)	Park et al. [35]
TFC	TFC: 109.4–211.0 ^6^	South America, Chile	Vega-Gálvez et al. [25]
TFC	TFC: 6.0 ^6^	South America, Peru	Drzewiecki et al. [26]
TFC	TFC (raw): 11.21 ^7^ TFC (roasted): 29.96 ^7^	South America, Argentina	Carciochi et al. [29]
TFC	TFC (raw): 11.06 ^7^ TFC (malted): 17.65 ^7^	South America, Argentina	Carciochi et al. [31]
TFC	TFC (freeze-dried samples): 504 ^7^ TFC (air-dried samples): 154 ^7^	South America, Colombia	Buitrago et al. [32]
TFC	TFC: 1.3 ^7^	Commercial	Hur et al. [45]
FFC, BFC	FFC (white): ≈60 ^6^ FFC (red): ≈175 ^6^ FFC (black): ≈150 ^6^ BFC (acd hdl): 40–150 ^6^ BFC (akl hdl): 65–160 ^6^	Commercial	Tang et al. [39]
Free flavonols Bound flavonols	Free flavonols: 30–113 ^8^ Bound flavonols: 16–147 ^8^	South America, Peru	Abderrahim et al. [24]

^1^ mg Gallic Acid Equivalents (GAE) 100 g^−1^ dm; ^2^ mg Gallic Acid Equivalents (GAE) 100 g^−1^ fw; ^3^ µmol GAE g^−1^ dm; ^4^ µg g^−1^ dm; ^5^ mg Rutin Equivalent (RE) 100 g^−^^1^; ^6^ mg Catechin Equivalent (CE) 100 g^−1^ dm: ^7^ mg Quercetin Equivalent (QE) 100 g^−1^ dm; ^8^ mg Quercetin Equivalent (QE) 100 g^−1^ fw; ↑: Increase; acd hdl: acid hydrolysis; akl hdl: alkaline hydrolysis; BFC: Bound Flavonoid Content; BPC: Bound Phenolic Content; FC: Folin–Ciocalteu assay; FFC: Free Flavonoid Content; FPC: Free Phenolic Content; PB: Prussian Blue assay; TFC: Total Flavonoid Content; TPC: Total Phenolic Content.

##### Profiling of Phenolic Compounds

Profiling methods based on High-Performance Liquid Chromatography (HPLC), gas chromatography (GC), liquid chromatography–mass spectrometry (LC-MS), and gas chromatography–mass spectrometry (GC-MS) have been developed in order to identify and quantify phenolic compounds in quinoa seeds. They enable one to overcome some of the drawbacks of the Folin–Ciocalteu assay, such as possible overestimation upon the reaction of non-polyphenolic components (e.g., tyrosine and tryptophan amino acids) and reducing sugars with the Folin–Ciocalteu reagent. Moreover, they allow for the quantification of as many phenolic components as possible in a single extract.

With respect to spectrophotometric methods, profiling methods have been scarcely applied for the characterization of phenolic compounds in quinoa seeds. Among phenolic acids, protocatechuic acid, *p*-hydroxybenzoic acid, vanillic acid, syringic acid, *p*-coumaric acid, ferulic acid, sinapic acid and isoferulic acid were identified in white and pigmented quinoa from China by HPLC [18]. Values were generally higher in black than white quinoa (Table 3). Interestingly, *p*-coumaric was the main phenolic acid in white quinoa, while syringic acid was the most abundant in pigmented genotypes.

In the Chinese quinoa cultivar Jinli-1, Han et al. identified gallic acid as the predominant compound present in the free form, while ferulic acid was found as the predominant compound in the bound form (Table 3). [16]. The latter result can be explained by the fact that hydroxycinnamic acid derivatives are covalently linked with arabinose sidechains of the cell wall polysaccharides and lignin.

Phenolics in quinoa samples from South America were detected by LC-MS and HPLC. In raw grains of quinoa from Peru [47], total phenolic content, obtained as the sum of individual phenolics, was 686.42 µg g^−1^ dm (Table 3). Phenolic acids accounted for about 22% of the total phenolics, with trans-*p*-coumaric acid being the most abundant. The effect of heat puffing on phenolic content was also studied, and a significant increase in total phenolics was observed (Table 3).

Lower values were found by Carciochi et al. in quinoa seeds from Argentina [29]. They identified *p*-hydroxybenzoic, vanillic, *p*-coumaric, and ferulic acids. Vanillic acid was the predominant phenolic acid, followed by ferulic acid (Table 3). The content of benzoic acid derivatives (e.g., *p*-hydroxybenzoic acid and vanillic acid) increased significantly after puffing. As for cinnamic acid derivatives (i.e., *p*-coumaric and ferulic acid), a marked increase in their content was observed after heating treatment at 145 °C, while a drastic decrease occurred at the highest temperatures. The effect of malting on the same samples was also studied [31], and it was found that total phenolics increased (Table 3).

In Puno and Titicaca varieties grown in Serbia, ferulic acid, 5-O-caffeoylquinic acid, gentisic acid, *p*-coumaric acid, and ellagic were found (Table 3) [23]. Ferulic acid was the most abundant in Puno seeds, while it was not quantified in Titicaca. Ellagic acid was dominant in Puno seeds, and the second most dominant—after ferulic acid—in Titicaca seeds, although the content did not vary significantly. The 5-O-caffeoylquinic acid concentration was comparable in the seeds of the two cultivars. *p*-Coumaric was higher in the Titicaca cultivar. Comparing these data with other studies, it emerged that the concentration of ferulic acid found in the Puno extract was similar to the values obtained by Tang et al. [39], while the values of *p*-coumaric acid concentration in the two varieties were lower than those observed by Tang et al. [39].

Pereira et al. studied white and pigmented quinoa samples available on the market in Peru and Spain by LC-MS [48]. Hydroethanolic extracts of black quinoa showed the highest content of total phenolics (Table 3). In ethanolic extracts of quinoa seeds purchased in local markets in Central Italy, 4′-geranyloxyferulic acid was found [49]. In both methanolic and hydrolyzed extracts of commercial red quinoa, 3-hydroxybenzoic acid, 4-hydroxybenzoic acid, protocatechuic acid, isoferulic acid, vanillic acid, and the flavonoid quercetin were identified by GC-MS, (Table 3) [50]. Vanillic was the most abundant phenolic acid in quinoa samples purchased in Seoul (Korea), while gallic and chlorogenic acids occurred in traces (Table 3) [45]. 

Carrasco-Sandoval et al. characterized the phenolic profile of ten quinoa seed samples from different varieties grown under different agronomic conditions by HPLC, after extraction with an ultrasound-assisted extraction method [51]. Total phenolics ranged from 1.10 to 1.99 mg kg^−1^ (Table 3), and caffeic, vanillic, t-ferulic acid and vanillin were only found in quantifiable concentrations.

Profiling methods also enabled the identification of individual components and the conjugated forms of flavonoids, such as monoglycosidic and diglycosidic derivatives.

A total of 11 flavonoids, including quercetin and kaempferol glycosides, were identified in quinoa seeds retrieved from the market [42]. Other compounds, such as acacetin, epicatechin, coumaryl malate, were also detected in small amounts. 

Quercetin and kaempferol, alongside their glucosides, were identified in all quinoa samples from South America, and the effect of processing on them was investigated [29,31,47]. In raw samples from Peru, flavonoids were the major fraction, and their content increased after puffing (Table 3) [47]. Quercetin 3-O-rutinoside was the main flavonoid both in raw and puffed seeds (Table 3) [47]. Roasting and malting also increased quercetin and kaempferol content in quinoa samples from Argentina (Table 3) [29,31].

Stikić et al. determined the flavonoid profile of Puno and Titicaca quinoa cultivars grown in Europe, using UHPLC–DAD MS/MS, and found that the most abundant flavonoid was rutin, for which a higher content was found in Titicaca seeds (Table 3) [23]. As for the concentration of quercetin and isorhamnetin-3-O-rutinoside, no statistically significant differences were found; quercetin-3-O-galactoside, naringin, and phlorizin concentrations were higher in the Titicaca cultivar; aesculin was higher in Puno seeds. The values obtained for quercetin concentrations were comparable to those obtained by Tang et al. [39]. 

Flavonoid glycosides (FG) were identified in samples retrieved from the market and from Chile by Graf et al. [52]. Comparable values were found in both sample categories (Table 3). Significant differences were observed among samples grown in different areas of Chile, possibly due to the effect of pedoclimatic conditions on flavonoid content. In detail, genotypes grown in northern Chile had a total flavonoid glycoside content 2.6-fold higher than those from central and southern Chile. The analysis of flavonoid profiles by HPLC-DAD-ESI/MS in a Brazilian genotype of white quinoa allowed for the six flavonol glycosides derived from quercetin and kaempferol to be determined [53]. Quercetin 3-O-(2″,6″-di-O-α-l-rhamnoside)-β-d-galactoside was the most abundant. The isoflavones genistein and daidzein were identified by HPLC in six quinoa samples from Chile [25]. The genistein content ranged between approximately 0.39 and 0.52 mg 100 g^−1^ dm, while daidzein varied from 0.60 to 1.93 mg 100 g^−1^ dm (Table 3).

#### 3.3.2. Betalains

Betalains are water-soluble pigments responsible for the color of plant tissues. They are categorized into betacyanins and betaxanthins. The former confers a red and violet color to plant tissues, while the latter provides a yellow shade. In addition to acting as pigments, they have a significant free-radical scavenging activity that is responsible for their beneficial health effects [54]. Their occurrence in food is rather limited: they have been identified in a selected few plants, namely red beet, cactus pears and amaranthus. More recently, they have also been detected in *Ullucus tuberosus*, a root crop of the Andean region of South America; in *Basella rubra*, a leafy vegetable; in *Opuntia ficus-indica* and *Opuntia stricta*, the prickly pear; in *Hylocereus polyrhizus*, the dragon fruit [55].

So far, betalains have been investigated in quinoa samples from South America and in commercial samples. Abderrahim et al. determined betalains in quinoa samples from the Peruvian Altiplano by spectrophotometric analysis and found total betalain values ranging between 0.15 and 6.10 mg 100 g^−1^ fw (Table 4) [24]. In samples from the same geographical area, several betalains were identified [56]. Amaranthin and its corresponding isomer iso-amaranthin were the most abundant betalains, and red-violet quinoa seeds were especially rich in them. Dopaxanthin, the betacyanin betanin and its isomer isobetanin were also detected in the varieties with red–violet grains. The varieties with yellow–orange seeds presented some betaxanthins: the main pigments were dopaxanthin and dopamine-betaxanthin. Proline-derived betaxantin was found in varieties with light-yellow colored seeds and in one black variety. In pigmented quinoa samples retrieved from the market in Ontario (USA), Tang et al. identified betanin and isobetanin [39].

#### 3.3.3. Carotenoids

Carotenoids are natural pigments conferring a peculiar yellow to orange–red color to plant tissue. They typically have a 40-carbon chain backbone composed of eight molecules of isoprene. Carotenes and xanthophylls are two major sub-groups of carotenoids. The former is made up of carbon and hydrogen atoms, while the latter are oxygenated carotenes.

Dietary intake of carotenoids has beneficial effects on human health, such as protection against cardiovascular diseases, cancer, and cataract and macular degeneration [57]. Food plays a pivotal role as a unique source of carotenoids, since they cannot be synthetized ex-novo by the human body.

Carotenoid content has been so far investigated in quinoa samples grown in Egypt (Africa), and Finland (Europe), as well as in commercial samples. β-Carotene and lycopene were determined in five genotypes of quinoa grown in Egypt (Table 5) [14]. A comparable amount of β-carotene was found also in a commercial sample of white quinoa [58], while higher values were observed in pigmented quinoa samples (Table 5). Six carotenoids were detected in quinoa samples grown in Finland [22]. All-trans-E-lutein was the main carotenoid identified, with neochrome A representing a distant second one (Table 5). The neochrome A content was comparable to zeaxanthin (0.22 mg kg^−1^) and the neochrome B content was 0.11 mg kg^−1^ (Table 5). Lutein isomers A and B contents were 0.09 and 0.21 mg kg^−1^, respectively.

Lutein was also the main carotenoid identified in both white and pigmented genotypes retrieved from the market [58]. Carotenoids were also detected in white and pigmented quinoa samples purchased in Ontario (Canada) [59], and pigmented varieties were richer in these components than white quinoa. The highest values were detected in black quinoa. Interestingly, the carotenoid content detected in black rice varieties was higher than in red genotypes, as well [33]. However, it is worth underlining that carotenoids were also detected in white quinoa samples, while white cereals, such as rice and corn, have no carotenoids.

#### 3.3.4. Tocols

Tocols encompass tocopherols and tocotrienols that are collectively referred to as vitamin E. From a chemical point of view, tocopherols are methylated phenols with a saturated side chain, while tocotrienols have an unsaturated isoprenoid side chain.

Tocopherols may exist in four isoforms, namely α-, β-, γ- and δ-tocopherol, which differ in the position of the methyl group in the chromanol ring. They also differ in their in vitro and in vivo antioxidant activities. Tocotrienols exist in four isoforms (α-, β-, γ- and δ-tocotrienol), as well.

So far, the content of tocols has been moderately investigated in quinoa seeds.

Tocols have not been studied in quinoa grown in African and Asian countries. As regards South America, four quinoa varieties grown in Peru (Blanca de Juli, Roja Pasankalla, Negra Collana and Amarilla de Maranganí) were studied by Pachari et al. [60]. The main tocopherol isomer was α-tocopherol, with values ranging between 463 and 1444 ppm (Table 5). The highest α-tocopherol and total tocopherol content was found in the Amarilla de Maranganì variety. Carciochi et al. determined the tocol content in quinoa samples grown in Buenos Aires province (Argentina) [30]. All four vitamers of tocopherols were identified. γ-Tocopherol was the most abundant, followed by α-tocopherol (Table 5). The content of β-tocopherol and δ-tocopherol was 0.9 and 3.1 mg kg^−1^ dm, respectively. Lower values of the isoforms α- and γ-tocopherols were detected in a white genotype quinoa sample developed and adapted to the tropical climate of Brazil (Table 5) [53].

Tocopherol and tocotrienol content was investigated in nine varieties of quinoa (pigmented and non-pigmented) from four countries, i.e., Bolivia, Colombia, Denmark and Peru [61]. Chromatographic analysis showed that the two isoforms of tocotrienol (α- and β-tocotrienols) and the β-isoform of tocopherol were absent in both pigmented and non-pigmented varieties. The three isoforms (α, β and γ) of tocopherol were present in different amounts (Table 5). It was observed that varieties with the greatest content in α-tocopherol, also had a higher vitamin E activity than others.

All four tocopherol homologues were identified in three commercial samples of white and pigmented quinoa seeds from a no specified region of Andes in South America [59]. γ-Tocopherol was the main homologue in both white and pigmented samples, with black and red cultivars showing a content approximately two-fold higher than that of the white sample (Table 5).

α-, γ- and δ-Tocoferol were identified in 39 samples of pigmented and non-pigmented quinoa [62]. γ-Tocopherol was the most abundant isomer, and black genotypes were richer than white varieties (Table 5). γ-Tocopherol was also the most abundant in the commercial quinoa samples studied by Niro et al. (Table 5) [58]. On the other hand, β-tocopherol was found in samples of commercial red quinoa (Table 5) [50].The bioaccessibility of β-tocopherol was also detected in the hydrolyzed extracts of quinoa and proved to be high (≈70%).

#### 3.3.5. Phytoecdysteroids

Phytoecdysteroids (PE) are a wide group of plant steroids whose structure is characterized by a steroid skeleton 5β-cholestanol containing a 6-ketone ring B and a hydroxyl group in position C-14α [63]. They occur as polar and non-polar conjugates, including esters of aliphatic and phenolic acids, sulfate and phosphate, ethers, and glycosides. Despite being widespread, a small percentage of plants contain detectable levels of PE [63]. The compound 20-Hydroxyecdysone (20 HE) is the most common phytoecdysteroid. Based on in-vitro and in-vivo studies, they are considered to play important activities in the human body. They act as anabolic, growth-promoting, antidiabetic, immunomodulatory, hepatoprotective, neuroprotective, hypocholesterolemic, wound healing, antidepressive and antioxidant agents [63].

Phytoecdysteroids have been poorly studied in quinoa. They have been identified in Chilean quinoa varieties and in commercial quinoa samples [52]. Their content, expressed as total phytoecdysteroid content, varied between 224 and 570 µg g^−1^ fw in Chilean varieties, and between 138 and 568 µg g^−1^ fw in commercial samples (Table 5). Chromatographic analysis showed that 20 HE amounts ranged between 184 and 491 µg g^−1^ fw.

### 3.4. Antinutritional Factors

Anti-nutritional factors (ANFs) are molecules that exert effects contrary to optimum nutrition. They can react with nutrients, thus interfering with their absorption [9]. Saponins, tannins, and phytic acid are the main ANFs occurring in quinoa [9].

#### 3.4.1. Saponins

Saponins are plant glucosides consisting of an aglycone unit linked to one or more sugars or oligosaccharides. The aglycone unit can be a triterpenoid (sapogenin) or a steroidal molecule (sapogenol) [64], and can be bound to D-galactose, L-arabinose, L-rhamnose, D-glucose, D-xylose, D-mannose, and D-glucuronic acid. Chains are commonly linear and include two to five saccharide units.

Saponin content is influenced by environmental factors: drought decreases sapogenin accumulation [65], while salinity increases sapogenin production [66].

These phytochemicals provide quinoa seeds with a bitter taste, however, processing methods, such as washing before cooking or mechanical dehulling by abrasion, enables their removal [67].

Saponins are commonly considered ANFs due to their hemolytic, membranolytic, and fungitoxic activities [68]. A significant reduction in potassium, iron, manganese and magnesium content was also reported [9]. Nevertheless, the nutritional role of these components is not unequivocal. Recently, a hypocholesterolemic effect was reported in experimental animals, due to saponin ability to inhibit cholesterol absorption from the intestinal lumen. In-vivo studies showed that legume saponins have been found to have a hypocholesterolemic effect and anticarcinogenic, antioxidative, antitumor, antivirus, antihepatic, antidiabetic, and hepatoprotective activities [69].

Saponin content and profile have been recently investigated in quinoa.

Saponin content was determined spectrophotometrically in quinoa grown in Egypt, China, and the USA, as well as in commercial samples. Samples grown in the USA [70] showed a total saponin content (TSC) higher than those cultivated in Egypt (Table 6) [14]. Among the samples from Egypt, the genotype Regalona exhibited the highest saponin content. Furthermore, the analysis allowed for the identification of one cultivar (i.e., kvlsra2) as the most ideal genotype for human consumption, as it possessed the lowest content of saponins and was consequently sweet and preferable. In a non-pigmented quinoa variety grown in China (Asia), the TSC was 15.50 mg oleanic acid equivalents (OAE) g^−1^ dm [16] (Table 6). In quinoa samples purchased in Spain, saponin content ranged between 2.6 and 55.1 g kg^−1^, depending on the extraction solvent (Table 6) [40].

The afrosimetric method was used to determine saponin content in four European quinoa cultivars grown in southern Germany in 2015 and 2016 [71]. The lowest value was observed in the sweet cultivar Jessie in both growing seasons (<0.7 mg g^−1^ in 2015 and 0.0 mg g^−1^ in 2016), while the varieties Puno, Titicaca and Zeno had a considerable amount of saponins (≈2.6–2.8 mg g^−1^ in 2015 and 2.8–3.5 mg g^−1^ in 2016). Quinoa samples grown in 2015 showed saponin values lower than those cropped in 2016, possibly because 2015 was drier than the 2016 growing season. Saponin values lower than 0.04 g 100 g^−1^ dm were generally found by Diaz-Valencia et al. in different varieties of red, black and white quinoa, except for a sample of a white quinoa cultivar (i.e., cv Syetetuba) which showed a saponin content of 3.4 g kg^−1^ dm (Table 6) [41].

The saponin profile has been also studied. Serjanic acid, oleanolic acid, phytolaccagenic acid and hederagenin were the four aglycones identified in commercial quinoa samples [50,72]. The highest total saponin (TS) was found in red quinoa extracts by Navarro del Hierro et al. and decreased to 20 g kg^−1^ upon extraction after hydrolysis of the sample (Table 6) [50]. No sapogenins were detected in the non-hydrolyzed quinoa extract, while the sapogenin content was 56.3 g kg^−1^ in the hydrolyzed extract (Table 6). 

Herrera et al. analyzed saponin-rich extracts of commercial red quinoa seeds to investigate the impact that acid hydrolysis can have on the content and profile of saponins (Table 6) [24]. Saponins were detected by HPLC-DAD-MS, and the sapogenin profile was obtained by GC-MS. 

Three main saponins were identified in quinoa seeds of a variety harvested in 2018 from an Argentinian farm [73]. Saponin C, with hederagenin as the aglycone, was found in a greater amount than saponin A and saponin B, which had phytolaccagenic acid as the aglycone (Table 6). Phytolaccagenic acid was the predominant sapogenin in quinoa varieties grown in Washington State (USA) [70]. Phytolaccagenic acid and hederagenin accounted for approximately 80 and 30% of the total saponins, respectively.

A total of 24 putative saponins were identified in quinoa commercial samples grown in Denmark and Bolivia [74]. It was observed that the Danish-bred variety Puno exhibited the highest diversity in saponins, while the variety Vikinga showed the lowest number of saponins. Upon GC–MS analysis, three main aglycones (i.e., oleanolic acid, hederagenin, and phytolaccagenic acid) were identified (Table 6). Hederagenin was generally the most abundant aglycone, followed by phytolaccagenic acid and oleanolic acid.

The 28-O-β-D-glucopyranosyl esters of oleanolic acid, serjanic acid, and phytolaccagenic acid, hederagenin substituted differently in C3, were identified in 29 varieties of quinoa grown in different areas of the Peruvian Altiplano and valleys, by HPLC-TOF/Q-TOF analysis [56].

Quantitative and qualitative determination of saponins in eight quinoa accessions from different origins and one commercial variety (Regalona Baer) of Chilean origin (control) was performed by De Santis et al. [67]. Total saponins, obtained from all aglycones (I–VII) of the whole seeds of quinoa samples, ranged from 0.05 to 2.00%, indicating a high variability. However, based on this parameter, it was observed that all accessions, but one, had a value lower than the control (Regalona Baer), which is a medium-bitter variety. From a qualitative point of view, the three major quinoa saponin aglycones identified were oleanolic acid, hederagenin and phytolaccagenic acid. The study highlighted that the investigated quinoa accessions had a low potential saponin content, likely because of specifically dry climatic conditions. Reguera et al. determined saponin content in three different varieties (Titicaca, Salcedo e Regalona) grown in three different sites (Chile, Peru and Spain) [75]. Saponin content varied between 8 and 13 g kg^−1^, but differences were not significantly different. Hence, it was suggested that this trait is regulated by the genotype more than by the environment.

#### 3.4.2. Tannins

Alongside saponins, tannins are anti-nutritional factors present in quinoa [9]. Their undesirable effect is due to their ability to complex proteins and macromolecules, such as starch, thus decreasing the nutritional value of food. They also provide an astringent taste and thus reduce the palatability of food. In the human body, they can damage intestinal mucosa and interfere with the absorption of iron, glucose and vitamin B12 [9].

From a chemical point of view, they are polyphenolic compounds and include several structures, from dimers to large polymers. The main sub-classes are condensed tannins and hydrolyzable tannins. The former are oligomers or polymers composed of flavan-3-ol units and can be classified as B-type or A-type, based on the interflavanic bond between the subunits. Hydrolyzable tannins encompass gallotannins and ellagitannins which can be hydrolyzed into gallic and ellagic acids, respectively [76].

Tannin content in quinoa seeds has been poorly investigated, so far. It has been reported that quinoa seeds contain a small amount of tannins and adequate processing may contribute to reducing their content [9]. A total amount of 0.88 mg CE g^−1^ was observed in quinoa seeds grown in Peru [26], while in samples from Africa, values ranged from 0.23 (Regalona genotype) to 0.31 mg g^−1^ dm (kvl-sra2 genotype) (Table 6) [14].

#### 3.4.3. Phytic Acid

Quinoa seeds also contain phytate, which is the major storage form of phosphorus in most plant seeds, and a mineral absorption inhibitor [77]. Phytic acid binds to positively charged divalent cations, such as iron, zinc, calcium, and proteins, and forms phytate complexes (InsP6) that are stable at intestinal pH, thus inhibiting the absorption of minerals in the small intestine. The degradation of phytate content is thus very important to decrease its negative effect on mineral bioavailability.

The content of phytic acid and its salt form has been poorly studied in quinoa seeds. Reguera et al. determined phytic acid content in three quinoa varieties grown in experimental fields located in three different sites (Spain, Peru and Chile) [75]. Total phytate was determined by the myo-inositol hexakisphosphate method, and values ranging from 2 to 4 g kg^−1^ were observed (Table 6). No differences in phytate content were found among cultivars grown in the same location, while the concentration of this anti-nutritional factor in a given genotype varied among locations. Hence, phytic acid content in quinoa seeds was mostly affected by environmental factors. The application of fermentation to decrease the phytic acid content has recently been investigated [78]. It was found that upon fermentation (both spontaneous and with *Lactobacillus plantarum* 299v^®^) phytate decreased by 72% (Table 6).

**Table 6:** Anti-nutritional factors in quinoa seeds.

Anti-Nutritional Factors	Analytical Method	Analyte Content	Quinoa Sample Origin	Reference
Saponins	Spectrophotometry	TSC: 2.76–4.12 ^1^	Africa, Egypt	Saad-Allah et al. [14]
Saponins	Spectrophotometry	TSC: 15.50 ^3^	Asia, China	Han et al. [16]
Saponins	Spectrophotometry	TSC: 6.6–30.9 ^1^	USA	Medina-Meza et al. [70]
Saponins	Spectrophotometry	TSC (ethanol:water): 44.3 ^1^ TSC (ethanol): 55.1 ^1^ TSC (water): 2.6 ^1^	Commercial	Navarro del Hierro et al. [40]
Saponins	Afrosimetric method	TSC: 0.0–3.5 ^1^	Europe, Germany	Präger et al. [71]
Saponins	Afrosimetric method	TSC: 0.0–3.4 ^1^	Multi-origin (South America, Peru and Brazil + Commercial)	Diaz-Valencia et al. [41]
Saponins	HPLC	TS (extract): 78.6 ^1^ TS (hydrolyzed extract): 20 ^1^ SC (extract): nd SC (hydrolyzed extract): 56.3 ^1^ Oleanolic acid (hydrolyzed extract): 15.5 ^1^ Hederagenin (hydrolyzed extract): 8.2 ^1^ Serjanic acid (hydrolysed extract): 18.3 ^1^ Phytolaccagenic acid (hydrolyzed extract): 14.4 ^1^	Commercial	Navarro del Hierro et al. [50]
Saponins	HPLC GC-MS	TS: 15.2 ^1^ Identified sapogenins: oleanolic acid, serjanic acid, and phytolaccagenic acid, hederagenin	Commercial	Herrera et al. [72]
Saponins	HPLC	Saponin A: 1.4–10 ^4^ Saponin B: 0.86–10 ^4^ Saponin C: 10–30 ^4^	South America, Argentina	Bonfiglio et al. [73]
Saponins	GC-MS	Phytolaccagenic acid: 16.72 ^1^ Hederagenin: 4.22 ^1^	USA	Medina-Meza et al. [70]
Saponins	GC-MS LC–MS/MS	Identification of 24 saponins Identification of three main aglycones	Multi-country (South America, Bolivia + Europe, Denmark)	Ruiz et al. [74]
Saponins	Afrosimetric method HPLC-TOF/Q-TOF	TSC: 2–83 ^5^ Identification of 28-O-β-D-glucopyranosyl esters of oleanolic acid, serjanic acid, and phytolaccagenic acid, hederagenin differently substituted in C3	South America, Peru	Escribano et al. [56]
Saponins	GC	TS: 0.05–2.00 ^6^ Identified sapogenins: oleanolic acid, hederagenin and phytolaccagenic acid	Multi-country (South America, Chile + USA)	De Santis et al. [67]
Saponin	Spectrophotometry	TSC: 8–13 ^1^	Multi-country (Spain, Peru, Chile)	Reguera et al. [75]
Tannins	Spectrophotometry	TNC: 0.88 ^2^	South America, Peru	Drzewiecki et al. [26]
Tannins	Spectrophotometry	TNC: 0.23–0.31 ^1^	Africa, Egypt	Saad-Allah et al. [14]
Phytic acid	Spectrophotometry	Phytate content: 2–4 ^1^	Multi-country (Spain, Peru, Chile)	Reguera et al. [75]
Phytic acid	HPLC	Phytate (raw seeds): 8.80 ^1^ Phytate (fermented seeds): 2.24 ^1^	Commercial	Castro-Alba et al. [78]

^1^ g kg^−^^1^; ^2^ mg Catechin Equivalents (CE) g^−1^ dm; ^3^ mg Oleanolic Acid Equivalents (OAE) g^−1^ dm; ^4^ mg 100 mL^−^^1^; ^5^ mm; ^6^ %; TNC: Tannin Content; TS: Total Saponins; TSC: Total Saponin Content.

## 4. Conclusions

Both hydrophilic and lipophilic functional components have been identified and quantified in quinoa seeds. Phenolic compounds are the most investigated functional component class. Determination by spectrophotometric assays was mainly performed. However, a number of studies have also reported on phenolic compound profiles. Betacyanins and phytoecdysteroids have been poorly investigated.

As far as anti-nutritional factors are concerned, saponins are the most studied. The interest in these components is possibly due to their contribution to quinoa bitterness and subsequent appreciation by consumers. In contrast, tannins and phytic acid have received little attention.

As regards the geographical origin of quinoa, seeds grown in South America have been mainly studied, and a more comprehensive characterization in terms of the functional and anti-nutritional components is available. However, in most studies no correlations between phytochemicals and factors such as quinoa variety, geographical origin and growing conditions, have been reported. Commercial samples have also been studied and data are available for inclusion in food composition databases. Validation and harmonization of analytical methods for the determination of functional components are desirable for the comparison of composition data.

## Figures and Tables

**Table 1:** Search strategy.

Search ID	Scopus Query	Documents (No.)
#1	(TITLE-ABS-KEY (quinoa) AND TITLE-ABS-KEY (antioxidants)) AND PUBYEAR > 2015	211
#2	(TITLE-ABS-KEY (quinoa) AND TITLE-ABS-KEY (saponins)) AND PUBYEAR > 2015	98
#3	(TITLE-ABS-KEY (quinoa) AND TITLE-ABS-KEY (flavonoids)) AND PUBYEAR > 2015	90
#4	(TITLE-ABS-KEY (quinoa) AND TITLE-ABS-KEY (phenolic AND compounds)) AND PUBYEAR > 2015	73
#5	(TITLE-ABS-KEY (quinoa) AND TITLE-ABS-KEY (bioactive AND compounds)) AND PUBYEAR > 2015	56
#6	(TITLE-ABS-KEY (quinoa) AND TITLE-ABS-KEY (phytochemicals)) AND PUBYEAR > 2015	37
#7	(TITLE-ABS-KEY (quinoa) AND TITLE-ABS-KEY (carotenoids)) AND PUBYEAR > 2015	30
#8	(TITLE-ABS-KEY (quinoa) AND TITLE-ABS-KEY (tocopherols)) AND PUBYEAR > 2015	28
#9	(TITLE-ABS-KEY (quinoa) AND TITLE-ABS-KEY (betalains)) AND PUBYEAR > 2015	11
#10	(TITLE-ABS-KEY (quinoa) AND TITLE-ABS-KEY (tannins)) AND PUBYEAR > 2015	13
#11	(TITLE-ABS-KEY (quinoa) AND TITLE-ABS-KEY (phytoecdysteroids)) AND PUBYEAR > 2015	5
#12	(TITLE-ABS-KEY (quinoa) AND TITLE-ABS-KEY (phytic acid)) AND PUBYEAR > 2015	17
	Total	669

**Table 3:** Phenolic compound profile in quinoa seeds.

Analyte	Analytical Method	Analyte Content	Quinoa Sample Origin	Reference
Phenolic acids	HPLC	Phenolic acids (white): 3.04–10.67 ^1^ Phenolic acids (red): 0.41–28.28 ^1^ Phenolic acids (black): 2.90–28.68 ^1^	Asia, China	Liu et al. [18]
Phenolic acids and flavonoids	HPLC	Gallic acid (free): 295.11 ^1^ Gallic acid (bound): nd Protocatechuic acid (free): 126.27 ^1^ Protocatechuic acid (bound): nd p-Hydroxybenzoic acid (free): 54.57 ^1^ p-Hydroxybenzoic acid (bound): 22.13 ^1^ Chlorogenic acid (free): 7.86 ^1^ Chlorogenic acid (bound): nd Vanillic acid (free): 26.87 ^1^ Vanillic acid (bound): 17.14 ^1^ Caffeic acid (free): 3.81 ^1^ Caffeic acid (bound): nd Syringic acid (free): 21.20 ^1^ Syringic acid (bound): 4.08 ^1^ Vanillin (free): 21.92 ^1^ Vanillin (bound): nd p-Coumaric acid (free): 48.39 ^1^ p-Coumaric acid (bound): 15.92 ^1^ Ferulic acid (free): 118.18 ^1^ Ferulic acid (bound): 72.62 ^1^ Rutin (free): 52.14 ^1^ Rutin (bound): 7.64 ^1^ Quercetin (free): 17.61 ^1^ Quercetin (bound): 6.51 ^1^ Total (free): 793.93 ^1^ Total (bound): 146.04 ^1^	Asia, China	Han et al. [16]
Phenolic acids	LC-MS	Total phenolic acids (raw): 151.43 ^1^ trans-p-coumaric acid (raw): 68.10 ^1^ Total phenolics (raw): 686.42 ^1^ Total phenolics (popped): 784.63 ^1^	South America, Peru	Paucar-Menacho et al. [47]
Phenolic acids	HPLC	Total phenolics (raw): 2.13 ^4^ p-OH-benzoic (raw): 0.22 ^4^ Vanillic acid (raw): 0.88 ^4^ p-Coumaric acid (raw): 0.09 ^4^ Ferulic acid (raw): 0.57 ^4^ Total phenolic acids (raw): 1.75 ^4^	South America, Argentina	Carciochi et al. [29]
Phenolic acids	HPLC	Total phenolics (raw): 2.13 ^4^ Total phenolic acids (raw): 1.75 ^4^ Total phenolics (malted): 16.7 ^4^ Total phenolic acids (malted): 15.0 ^4^	South America, Argentina	Carciochi et al. [31]
Phenolic acids	UHPLC–DAD MS/MS	Ferulic acid: nd—56.21 ^2^ 5-O-caffeoylquinic acid: 2.14–2.19 ^2^ Gentisic acid: nd—0.62 ^2^ p-Coumaric acid: 2.03–6.46 ^2^ Ellagic acid: 12.03–14.29 ^2^ Total phenolic acids: 23.51–72.46 ^2^	Europe, Serbia	Stikić et al. [23]
Total phenolics	LC-MS	Total phenolics (black): 574.7 ^3^ Total phenolics (red): 358.7 ^3^ Total phenolics (white): 483.4 ^3^	Commercial	Pereira et al. [48]
Phenolic acids	UHPLC-DAD	4′-geranyloxyferulic acid: 2.01 ^1^	Commercial	Fiorito et al. [49]
Phenolic acids	LC-MS	Vanillic acid: 1.3 ^2^ Gallic acid: 0.01 ^2^ Chlorogenic acid: 0.002 ^2^	Commercial	Hur et al. [45]
Phenolic acids and flavonoids	GC-MS	3-Hydroxybenzoic acid (free): 20 ^4^ 4-Hydroxybenzoic acid (free): 30 ^4^ Vanillic acid (free): 60 ^4^ Protocatechuic acid (free): 130 ^4^ Isoferulic acid (free): 70 ^4^ Quercetin (free): 160 ^4^ 3-Hydroxybenzoic acid (bound): 20 ^4^ 4-Hydroxybenzoic acid (bound): 60 ^4^ Vanillic acid (bound): 190 ^4^ Protocatechuic acid (bound): 90 ^4^ Isoferulic acid (bound): 100 ^4^ Quercetin (bound): 350 ^4^	Commercial	Navarro del Hierro et al. [50]
Phenolic acids	HPLC	Total phenolics: 1.10–1.99 ^2^ Caffeic acid: 0.14–0.33 ^2^ Vanillic acid: 0.34–0.95 ^2^ Vanillin: nd—0.09 ^2^ t-Ferulic acid: 0.21–1.03 ^2^	Not specified	Carrasco-Sandoval et al. [51]
Flavonoids	HPLC-ESI-MS	Identification of 11 flavonoids (i.e., quercetin and kaempferol glycosides)	Commercial	Balakrishnan et al. [42]
Flavonoids	LC-MS	TF (raw): 534.99 ^1^ TF (popped): 674.58 ^1^ Quercetin 3-O-rutinoside (raw): 73.33 ^1^ Quercetin 3-O-rutinoside (popped): 112.60 ^1^	South America, Peru	Paucar-Menacho et al. [47]
Flavonoids	HPLC	Quercetin (raw): 0.23 ^4^ Kaempferol (raw): 0.15 ^4^ Total flavonoid (raw): 0.37 ^4^ Quercetin (roasted at 190 °C): 1.98 ^4^ Kaempferol (roasted at 190 °C): 2.00 ^4^ Total flavonoid (roasted at 190 °C): 3.98 ^4^	South America, Argentina	Carciochi et al. [29]
Flavonoids	HPLC	Quercetin (raw): 0.23 ^4^ Kaempferol (raw): 0.15 ^4^ Total flavonoid (raw): 0.37 ^4^ Quercetin (malted): 1.36 ^4^ Kaempferol (malted): 0.27 ^4^ Total flavonoid (malted): 1.63 ^4^	South America, Argentina	Carciochi et al. [31]
Flavonoids	UHPLC–DAD MS/MS	Quercetin (Puno): 4.78 ^2^ Quercetin (Titicaca): 4.78 ^2^ Isorhamnetin (Puno): 3.00 ^2^ Isorhamnetin (Titicaca): nd Quercetin−3-O-galactoside (Puno): 1.09 ^2^ Quercetin−3-O-galactoside (Titicaca): 1.87 ^2^ Isorhamnetin−3-O-rutinoside (Puno): 2.37 ^2^ Isorhamnetin−3-O-rutinoside (Titicaca): 2.42 ^2^ Rutin (Puno): 30.1 ^2^ Rutin (Titicaca): 33.8 ^2^ Naringin (Puno): 0.07 ^2^ Naringin (Titicaca): 0.12 ^2^ Aesculin (Puno): 0.53 ^2^ Aesculin (Titicaca): 0.46 ^2^ Phlorizin (Puno): 0.08 ^2^ Phlorizin (Titicaca): 0.14 ^2^ Eriodictyol (Puno): 0.33 ^2^ Eriodictyol (Titicaca): nd	Europe, Serbia	Stikić et al. [23]
Flavonoid glycosides	HPLC-UV-MS	FG (Chile): 192–804 ^5^ FG (commercial): 196–674 ^5^	Multi-origin (Commercial + South America, Chile)	Graf et al. [52]
Flavonoid glycosides	HPLC-DAD-ESI/MS	Quercetin 3-O-(2″,6″-di-O-α-l-rhamnoside)-β-d-galactoside: 93.5 ^6^ Quercetin 3-O-(2″-O-β-apioside-6″-O-α-rhamnoside)-β-galactoside: 58.2 ^6^ Kaempferol 3-O-(2″,6″-di-O-α-rhamnoside)-β-galactoside: 23.73 ^6^ Kaempferol 3-O-(2″,6″-di-O-α-rhamnoside)-β-glucoside: 21.5 ^6^ Quercetin 3-O-rutinoside: 5.26 ^6^ Kaempferol 3-O-rutinoside: 5.28 ^6^	South America, Brazil	Sampaio et al. [53]
Isoflavones	HPLC	Genistein: 0.39–0.52 ^4^ Daidzein: 0.60–1.93 ^4^	South America, Chile	Vega-Gálvez et al. [25]

^1^ µg g^−1^ dm; ^2^ mg kg^−1^ dm; ^3^ µg mL^−^^1^; ^4^ mg 100 g^−1^ dm; ^5^ µg g^−1^ fw; ^6^ mg g^−1^ dm; nd: not detected; BFC: Bound Flavonoid Content; FCM. Folin-Ciocalteu method; FFBB: Fast Blue BB diazonium method; FFC: Free Flavonoid Content; FG: Flavonoid glycosides; TF: Total Flavonoids.

**Table 4:** Betalains in quinoa seeds.

Analytical Method	Analyte Content	Quinoa Sample Origin	Reference
Spectrophotometry	Total betalains: 0.15–6.10 ^1^	South America, Peru	Abderrahim et al. [24]
HPLC	Amaranthin: 0.8–148.6 ^2^ iso-Amaranthin: 0.8–145.5 ^2^ Betanin: 0.6–9.7 ^2^ iso-Betanin: 0.6–7.7 ^2^ Dopaxanthin: 0.4–85.3 ^2^ Dopamine-BX: 0.5–10.5 ^2^ Proline-BX: 0.3–7.5 ^2^	South America, Peru	Escribano et al. [56]
LC-MS	Identification of betanin and isobetanin	Commercial (red and black quinoa)	Tang et al. [39]

^1^ mg 100 g^−1^ fw; ^2^ mg kg^−1^ dm; BX: betaxanthin.

**Table 5:** Lipophilic non-nutrient functional components in quinoa seeds.

Functional Component Group	Analytical Method	Analyte Content	Quinoa Sample Origin	Reference
Carotenoids	Spectrophotometry	β-carotene: 10.5–12.65 ^1^ Lycopene: 1.48–2.40 ^1^	Africa, Egypt	Saad-Allah et al. [14]
Carotenoids	HPLC-APCI-MS/MS	all-trans-E-lutein: 1.47 ^1^ all-trans-zeaxanthin: 0.22 ^1^ Lutein isomer A: 0.09 ^1^ Lutein isomer B: 0.21 ^1^ Neochrome A: 0.24 ^1^ Neochrome B: 0.11 ^1^	Europe, Finland	Multari et al. [22]
Carotenoids	HPLC	Lutein (white): 85.6 ^2^ Lutein (pigmented): 265.2 ^2^ Zeaxanthin (white): 11.2 ^2^ Zeaxanthin (pigmented): 13.2 ^2^ Β-carotenone (white): 12.3 ^2^ Β-carotenone (pigmented): 23.60 ^2^ TC (white): 109.1 ^2^ TC (pigmented): 302.0 ^2^	Commercial	Niro et al. [58]
Carotenoids	Spectrophotometry HPLC	TC (white): 11.87 ^1^ TC (red): 14.97 ^1^ TC (black): 17.61 ^1^ all-trans-lutein (white): 7.56 ^1^ all-trans-lutein (red): 9.49 ^1^ all-trans-lutein (black): 11.32 ^1^ all-trans-zeaxanthin (white): 0.39 ^1^ all-trans-zeaxanthin (red): 0.47 ^1^ all-trans-zeaxanthin (black): 0.55 ^1^	Commercial	Tang et al. [59]
Tocols	HPLC	α-tocopherol (Blanca de Juli): 639 ^3^ α-tocopherol (Roja Pasankalla): 608 ^3^ α-tocopherol (Negra Collana): 463 ^3^ α-tocopherol (Amarilla de Marangani): 1444 ^3^ TT (Blanca de Juli): 1354 ^3^ TT (Roja Pasankalla): 1450 ^3^ TT (Negra Collana): 1512 ^3^ TT (Amarilla de Maranganì): 2275 ^3^	South America, Peru	Pachari et al. [60]
Tocols	HPLC	γ-tocopherol: 40.6 ^1^ α-tocopherol: 28.3 ^1^ β-tocopherol: 0.9 ^1^ δ-tocopherol: 3.1 ^1^	South America, Argentina	Carciochi et al. [30]
Tocols	HPCL-FL	α-tocopherol: 0.919 ^4^ γ-tocopherol: 2.67 ^4^ TT: 3.59 ^4^	South America, Brazil	Sampaio et al. [53]
Tocols	HPLC	α-tocopherol (non-pigmented): 39.41–53.27 ^1^ α-tocopherol (pigmented): 35.55–82.87 ^1^ β-tocopherol: nd γ-tocopherol (non-pigmented): 24.77–86.47 ^1^ γ-tocopherol (pigmented): 40.13–78.98 ^1^ δ-tocopherol (non-pigmented): 0.62–3.72 ^1^ δ-tocopherol (pigmented): 0.91–4.59 ^1^ α-tocotrienol: nd β-tocotrienol: nd Vit. E activity: 43.50–86.89 ^1^	Multi-country (Bolivia, Colombia, Denmark and Peru)	Granda et al. [61]
Tocols	LC-MS	γ-tocopherol (white): 25.89 ^1^ γ-tocopherol (red): 43.51 ^1^ γ-tocopherol (black): 46.90 ^1^ β-tocotrienol (white): 0.78 ^1^ β-tocotrienol (red): 0.82 ^1^ β-tocotrienol (black): 0.86 ^1^ Total Vit E (white): 37.49 ^1^ Total Vit E (red): 55.25 ^1^ Total Vit E (black): 59.82 ^1^	Commercial	Tang et al. [59]
Tocols	HPLC	γ-tocoferol (white): 839 ^2^ γ-tocoferol (red): 1210 ^2^ γ-tocoferol (black): 1619 ^2^ TT (white): 971 ^2^ TT (red): 1388 ^2^ TT (black): 1764 ^2^	Commercial	Pereira et al. [62]
Tocols	HPLC	α-tocopherol: 2.86 ^4^ β-tocopherol: 0.11 ^4^ γ-tocopherol: 5.90 ^4^ δ-tocopherol: 0.22 ^4^ α-tocotrienol: nd β-tocotrienol: traces γ-tocotrienol: traces δ-tocotrienol: nd	Commercial	Niro et al. [58]
Tocols	GC-MS	β-tocopherol (free): 0.51 ^1^ β-tocopherol (bound): 0.68 ^1^	Commercial	Navarro del Hierro et al. [50]
Phytoecdysteroids	LC-MS	TPE (Chile): 224–570 ^5^ TPE (commercial): 138–578 ^5^ 20 HE: 184–491 ^5^	Multi-origin (South America, Chile + Commercial)	Graf et al. [52]

^1^ mg kg^−^^1^ dm; ^2^ µg 100 g^−^^1^ dm; ^3^ ppm; ^4^ mg 100 g^−1^ fw; ^5^ µg g^−1^ fw; 20 HE: 20-Hydroxyecdysone; nd: not detected; PE: Phytoecdysteroids; TPE: Phytoecdysteroids; TT: Total Tocopherols.
